# A Preliminary Study of the Impacts of Duckweed Coverage during Rice Growth on Grain Yield and Quality

**DOI:** 10.3390/plants13010057

**Published:** 2023-12-23

**Authors:** Jingsheng Luo, Shaowu Hu, Tong Li, Fuhao He, Chao Tian, Yu Han, Yulin Mao, Liquan Jing, Lianxin Yang, Yunxia Wang

**Affiliations:** 1 Key Laboratory of Arable Land Quality Monitoring and Evaluation, Ministry of Agriculture and Rural Affairs, Yangzhou University, Yangzhou 225127, China; mx120210692@stu.yzu.edu.cn (J.L.);; 2 College of Environmental Science and Engineering, Yangzhou University, Yangzhou 225127, China; 3 Key Laboratory of Crop Genetics and Physiology of Jiangsu Province, Yangzhou University, Yangzhou 225009, China; 4 Co-Innovation Center for Modern Production Technology of Grain Crops of Jiangsu Province, Yangzhou University, Yangzhou 225009, China

**Keywords:** duckweed (*Lemna minor* L.), rice (*Oryza sativa* L.), yield, quality

## Abstract

The overuse and misuse of fertilizers have been causing duckweed outbreaks in irrigation ditches and paddy fields in many rice-growing areas. However, how duckweed coverage in a paddy field affects the rice yield and grain quality is under debate because duckweed may act as either a weed, competing with rice for mineral nutrients, or a “nutrient buffer”, providing significant ecological and economic benefits. To understand the effects of duckweed coverage throughout rice growth on the yield and quality of rice grains, an experiment with three *Japonica* rice cultivars was conducted with fertile lotus-pond bottom soil as a growth medium to provide sufficient mineral nutrients for both the duckweed and rice. Averaged across three rice cultivars, duckweed coverage decreased the panicle density but increased the spikelet density and grain weight, resulting in no significant change in the rice yield. Duckweed coverage had no impact on the processing and appearance quality in general, but significant duckweed-by-cultivar interactions were detected in the head rice percentage and grain chalkiness, indicating different sensitivities of different cultivars in response to the duckweed treatment. The decrease in breakdown and increase in setback values in the rapid visco analyzer (RVA) profile of rice flour suggested that duckweed coverage during rice growth worsened the cooking quality of the rice. However, no significant change in the palatability of the cooked rice was found. The most profound change induced by the duckweed was the nutritional quality; duckweed coverage increased the protein concentration but decreased the concentrations of Mg, Mn, Cu, and Zn in rice grains. This preliminary study suggested that duckweed coverage during rice growth has profound effects on the rice nutrient uptake and grain nutritional quality under the circumstances, and further research on the responses of the rice quality to the duckweed coverage in paddy fields in multiple locations and years is needed.

## 1. Introduction

Common duckweed (*Lemna minor* L.) is a small floating aquatic plant; its particular anatomical and physiological features enable it to grow in nutrient-rich or eutrophic freshwater environments, such as ponds, ditches, slowly flowing streams, and rice paddies [1,2]. As a primary producer, duckweed is a food source for fish, waterfowl, and small invertebrates and provides habitat for small organisms; thus, it has its unique ecological significance in the aquatic environment [3,4]. Owing to its fast growth and ability to tolerate and accumulate high concentrations of nitrogen (N), phosphorus (P), and heavy metals, duckweed has been used in wastewater treatment systems and ecological ponds to recycle N, P, and heavy metals from wastewater [5,6,7].

Before chemical fertilizers were widely used in China, duckweed had been introduced to paddy fields as a green manure to increase the soil fertility [8,9]. However, the continuously increasing application of chemical fertilizers to raise yields [10] has led to eutrophication from agricultural drainage [11]. Nitrogen and P losses through runoffs from rice paddy fields in China were estimated to reach 272.6 ± 101.2 Gg N yr^−1^ and 17.0 ± 6.4 Gg P yr^−1^, respectively [12]. The overuse and misuse of fertilizers have also resulted in the increasing incidence of duckweed outbreaks in irrigation ditches and paddy fields [13]. The full duckweed coverage of the water’s surface in a paddy during the early rice-growth stage could inhibit tiller formation, resulting in yield loss [14]. Farmers apply herbicides [15] or rice hull to control duckweed [16,17]. Hence, duckweed is treated as a damaging weed in farming practice.

Despite the negative effects of inhibiting rice tiller formation, the positive effects of duckweed in rice paddies have also been reported. As the most used N fertilizer, urea quickly converts to ammonium after entering the paddy fields, causing a large amount of ammonia volatilization and N loss [18]. Duckweed coverage in a paddy field, particularly at the early rice-growth stage, forms a physical barrier that directly blocks ammonia volatilization, allowing relatively more N to be retained in the surface water and soil for the growth of the duckweed and rice [19,20]. In addition, duckweed coverage decreases the water temperature and pH, which slows ammonia volatilization, especially at the early growth stage of rice [19,21,22]. At the middle and late growth stages, rice-leaf shading accelerates duckweed decomposition, releasing N and other minerals into the rice paddy [23]. Therefore, duckweed coverage could increase the nitrogen-use efficiency by decreasing N loss through volatilization or runoffs, showing significant ecological and economic benefits [19,20,22].

Different from rice straw, duckweed has a protein content that reaches 45% of its dry matter [24], with a low C/N ratio of only 8 [25]. A low C/N ratio may allow the N released from duckweed decomposition to not only fulfil the N requirement for microbial reproduction but also provide N for rice growth. This may have the same role as the N fertilizer applied at the panicle initiation practiced by farmers to increase the spikelet number and, thereby, increase the yield. In a recent report, a rice yield increase of 10.8–23.7% by duckweed coverage in a paddy field was attributed to the increase in the panicle size or grain number per panicle [26]. In addition, duckweed coverage in a paddy field prevented weed growth, especially in the early rice season [26], because the duckweed coverage blocked the sunlight and impeded the photosynthesis of weeds. This non-chemical weed-control strategy may provide an environmentally friendly way to improve the nutrient utilization of rice.

Although a number of studies have investigated the effects of the duckweed coverage in paddies on the rice growth and yield, no consensus has been reached because positive [19,20], negative [14], and no significant effects [27,28] of duckweed on the rice yield have been reported (Table S1). The effects of the duckweed treatment on the rice yield seem to be dependent on soil types [29], duckweed species [26], and fertilizer applications [22]. Duckweed coverage significantly increased the rice yield by 13.2% and 16.9% when urea and biogas slurry were applied, respectively; however, no significant yield increase was found when slow-release fertilizer or null fertilizer was applied [22]. This suggests that duckweed–rice interactions vary with the nutrient availability in the growth environment. In all eight reports on duckweed–rice interactions, three reported the rice cultivar name used in the experiment, and all three were different cultivars; hence, the lack of consensus may also be partially due to different rice cultivars used in different experiments.

To the best of our knowledge, there is only one publication on the rice-quality response to duckweed coverage to date, and only four quality traits were measured [27]. However, grain quality is an important aspect of rice production because the demand for high-quality rice will continue to rise in the near future owing to population growth and rising living standards in most rice-consuming countries [30]. To understand the effects of duckweed coverage throughout rice growth on the grain yield and quality of the rice, especially the nutritional quality, an experiment with three *Japonica* rice cultivars was conducted. Instead of common soil used in rice cultivation, we used fertile lotus-pond bottom soil to provide sufficient mineral nutrients for both the duckweed and rice and to simulate the situation of the organic fertilizer application that caused duckweed outbreaks in paddy fields near livestock farms. Our objective was to determine if duckweed competes with rice plants for mineral nutrients, resulting in a lower nutrient concentration in rice grains and, if applicable, to identify the basis for any observed differences in the duckweed turnover, rice-root environmental change, and uptake of mineral elements by the different rice cultivars. The results from this preliminary experiment will pave the way for large-scale field experiments with more cultivars in different environments.

## 2. Results

### 2.1. Yield and Yield Components

The duckweed coverage of the water’s surface started from the rice transplantation and continued to the middle grain-filling stage of the rice growth. Averaged across the three rice cultivars, duckweed coverage had no significant effect on the rice yield, but it affected the yield components in different directions (Table 1). Duckweed coverage significantly increased the spikelet number per panicle by 8.2% on average, with Jinxiangyu 1 (JXY1) being increased by 18.5%. Duckweed coverage also increased the grain weight by 2.1%, with JXY1 showing the highest increase among the three cultivars. However, duckweed coverage significantly decreased the panicle number per plant by 10.1%, averaged across the three rice cultivars, with the same decreasing trend for all the cultivars (Table 1). A decreasing trend in the filled-grain percentage (*p* = 0.061) was detected for the duckweed coverage.

### 2.2. Grain Quality

#### 2.2.1. Processing and Appearance Qualities

Duckweed coverage did not affect the processing and appearance qualities of the rice grains in general; however, among the eight parameters for assessing the processing and appearance qualities of the rice grains, three parameters showed significant duckweed-by-cultivar interactions: they were the head-rice percentage, chalky-grain percentage, and chalkiness degree (Table 2). Duckweed increased the head-rice percentage of Yangnongxiang 28 (YNX28) by 21.4% but decreased the head-rice percentages of JXY1 and Nanjing 9108 (NJ9108) by 10.5% and 13.3%, respectively. Duckweed coverage increased the chalky-grain percentage by 9.7% in JXY1 and 9.3% in YNX28 but had no significant effect on that in NJ9108 (Table 2). Duckweed coverage increased the chalkiness degree by 15.8% in YNX28 but had no significant effect on the chalkiness degree in the other two rice cultivars. The green-grain percentage refers to the weight ratio of the immature grains in brown rice. On average, duckweed coverage tended to increase the green-grain percentage in all three rice cultivars.

#### 2.2.2. Cooking and Eating Qualities

The cooking and eating qualities were assessed based on the RVA profile of milled rice flour (Table 3) and palatability of cooked rice (Table 4). The RVA profile evaluates the starch-pasting properties of milled rice flour. Among the eight parameters in the RVA profile, only three parameters were significantly affected by the duckweed treatment: they were the breakdown, setback, and peak time (Table 3). Averaged across the three rice cultivars, duckweed coverage significantly decreased the value of the breakdown by 5.9% but increased the value of the setback by 10.6%. The peak time for the duckweed treatment was significantly longer than that for the control. Among three rice cultivars, the effects of duckweed were statistically significant only in YNX28; this cultivar also had the highest breakdown and the lowest setback values (Table 3).

The palatability was characterized based on the hardness, stickiness, luster, balance degree, and overall palatability index of the cooked rice (Table 4). The three *Japonica* rice cultivars showed similar palatability indices, and the duckweed coverage treatment had no effect on the palatability traits of the rice grains.

#### 2.2.3. Nutritional Quality

Duckweed coverage increased the protein concentration in brown rice, with all the cultivars showing a similar increasing trend (Table 5). Averaged across the three cultivars, duckweed coverage decreased the concentrations of copper (Cu), manganese (Mn), and zinc (Zn) by 33.3%, 8.6%, and 19.5%, respectively (Table 5). Within the four macronutrients of calcium (Ca), potassium (K), magnesium (Mg), and phosphorus (P), only the concentration of Mg was significantly decreased by the duckweed coverage. Duckweed coverage significantly decreased the concentrations of Cu and Zn in the brown rice of NJ9108 by 48.8% and 32.6%, respectively. Duckweed coverage significantly decreased the concentrations of Cu and Mn by 32.1% and 24.3% but increased the K concentration by 9.4% in JXY1. For the element concentrations in brown rice, a significant duckweed-by-cultivar interaction was only detected for the Mn concentration: a significant decrease in JXY1 but no change in the other two cultivars.

The total contents of the elements accumulated in brown rice can be calculated by multiplying the brown-rice elemental concentration by the brown-rice yield (Table 6). On average, duckweed coverage significantly reduced the element contents of Mn, Cu, Zn, and Mg in brown rice by 16.0%, 37.3%, 25.1%, and 13.5%, respectively. The effects of the duckweed coverage on the total element content of brown rice were not consistent among the three cultivars. Duckweed coverage significantly decreased the element contents of P, K, and Mg by 21.7%, 21.2%, and 22.2% in YNX28, respectively. As for NJ9108, the contents of Cu and Zn significantly decreased by 55.6% and 42.3% for duckweed coverage, respectively. These substantial decreases in Cu and Zn contents by more than 40% were due to the combined reductions in the brown-rice yield (15.7%) and the concentrations of Cu (48.8%) and Zn (32.6%). By contrast, the contents of five out of the eight elements in brown rice showed an increasing tendency owing to duckweed coverage in JXY1, including P, K, Ca, Mg, and iron (Fe). However, a significant decrease in the Mn content was detected despite an increasing tendency in the brown-rice yield of JXY1 in response to the duckweed coverage treatment.

## 3. Discussion

Although duckweed coverage during the rice-growth season had clear effects on soil environments, as shown by the lower redox potential (Eh) and temperature (Figures S1–S3), its effect on the rice yield was relatively small (Table 1). Because duckweed coverage had different effects on yield components, it decreased the tiller formation but increased the spikelet formation; the tradeoff between the yield components led to little change in the final yield. Among the three *Japonica* rice cultivars, JXY1 was the most sensitive to the duckweed coverage. Duckweed coverage decreased the panicle number per plant in JXY1 by 12.1% but increased the spikelet number per panicle and single-grain weight by 18.5% and 3.8%, respectively. This indicates that the duckweed had adverse effects on the rice at the early-growth stage but had beneficial effects at the late-growth stage, even under the circumstance of growing in the fertile pond-bottom soil, which was rich in nutrients. This result agrees with those in a previous report by Wang et al. [26], where the authors found trends in rice-tillering inhibition and spikelet enhancement caused by the duckweed coverage in a paddy field. The tillering inhibition was attributed to the nutrient competition between the duckweed and rice seedlings [14] or the suppression in the tiller-bud outgrowth induced by the duckweed shading [26].

RVA parameters are used to assess the starch-pasting properties of milled rice flour. Rice with a higher breakdown value and a lower setback value usually has a better cooking quality [31]. Therefore, the significant decrease in the breakdown value and increase in the setback value caused by the duckweed coverage in the present study suggest that the presence of duckweed negatively impacts the cooking quality of the rice (Table 3). In addition, the increase in the grains’ protein concentration (Table 5) also indicated a decline in the cooking quality because a previous study found that the grains’ protein concentration was positively correlated with the setback value but negatively correlated with the breakdown value [32]. In general, duckweed coverage did not affect the processing and appearance qualities of the rice grains, and the most prominent change in the rice quality was in the nutritional quality. In the following sections, we will focus on discussing the reasons behind this change.

### 3.1. Elemental Dynamics of Rice as Affected by Duckweed Turnover

To ensure a good start to the plants’ establishment, the common practice in rice cultivation allocates a large portion of the total fertilizers in the early growth of rice as basal fertilizer and tillering fertilizer, which give sufficient nutrients for both duckweed and rice growth. But in areas with limited fertilizer input, competition between duckweed and rice seedlings may occur, which affects the rice growth, tiller formation, and final yield [14]. In a recent report, duckweed coverage significantly increased the rice yield by 13.2% and 16.9% when urea and biogas slurry were applied, respectively; however, the yield increase was smaller and non-significant when slow-release fertilizer or no fertilizer was applied [22]. Unfortunately, neither the tiller formation nor the panicle number was reported in other studies (Table S1), except for a study by Wang et al. [26]. A tiller reduction trend caused by the duckweed coverage was also observed for each cultivar in the present study. And the concentrations of several nutrients, including both macro- and micronutrients, were significantly decreased in the rice seedlings, averaged across the three cultivars (Table S2). For example, duckweed, a heavy-metal scavenger, reduced the macronutrients of N, K, Ca, and Mg by 18.0%, 11.2%, 33.4%, and 24.1%, respectively. The micronutrients of Mn, Cu, and Zn were also reduced by 42.2%, 22.0%, and 25.1%, respectively. This suggests that duckweed competed with rice seedlings for nutrients; meanwhile, the lower soil temperature (Figures S1 and S2) caused by the duckweed coverage might also inhibit the roots’ nutrient uptake.

During the stem-elongation stage, the rice canopy’s closure gradually blocked light from the duckweed, especially from the lower fronds of the duckweed cover. They senesced rapidly, sank to the soil surface, and decomposed with the help of microbes in the soil. Through the decomposition, a large amount of nutrients, such as N, P, and K, are released from the dying duckweed into the water [23]. At the rice-flowering stage, almost no green duckweed was observed on the water’s surface, which was also supported by no temperature difference in the soil between the control and duckweed treatment (Figures S1 and S2, respectively). The bio-availability of the nutrients released from the decomposing duckweed litter varied for different elements, with macronutrients being more mobile than micronutrients in the paddy water [23].

These macronutrients were taken up and assimilated by the rice plants and finally transferred to the grains, which may explain the insignificant decrease in the concentrations of P, K, and Ca (Table 5). In fact, an increasing trend in the protein concentration was detected for all three cultivars. This may be due to the duckweed coverage inhibiting NH_4_^+^ emission, resulting in relatively more N being assimilated by vegetation, including rice and duckweed, when compared with duckweed-coverage-free plots. Several reports have demonstrated that duckweed coverage reduced NH_4_^+^ emission directly by a physical barrier [19,20,29] or indirectly through lowering the water temperature and pH [19,22,29]. The total N uptake in rice plants and nitrogen-fertilizer-use efficiency were improved by the duckweed coverage in these previous reports [19,20].

We attributed the higher grain protein concentration in the duckweed treatment to reduced NH_3_ losses in the early rice-growth stage and the adequate N supply to the rice in the late-growth stage. Through decomposition, the duckweed released N for rice uptake throughout the rice-growth season, in particular, during the grain-filling period. In this manner, duckweed served as green manure for a continuous supply of N. Some researchers have suggested that no competition for N between duckweed and rice plants exists when adequate fertilizers are applied [19] because no yield reduction was found in duckweed plots. Our results suggested that N competition occurred at the early rice-growth stage but that duckweed decomposition returned N to the rice at the late-growth stage, as demonstrated by a substantial decrease in the N concentration in seedlings (Table S2) but an increase in the protein in grains (Table 5). The high percentage of protein in the solute of the decomposing duckweed plants could be the source for this phenomenon. Roots are capable of directly taking up and metabolizing organic N forms, including amino acids. In some cases, positively charged amino acids dominate soil-diffusive nitrogen fluxes [33]. Rice is notoriously poor in positively charged lysine, while duckweed is rich in this amino acid [34]. The rich presence of lysine emanating from decomposed duckweed at the late-growth stage of the rice might influence the lysine concentration in the grain; this interesting question merits further investigation.

### 3.2. Elemental Dynamics of Rice as Affected by Duckweed-Induced Rhizospherical Changes

The decreases in the concentrations of the micronutrients in rice grains, caused by the duckweed treatment, may be related to changes in soil factors. The continuous cooling effect of the duckweed coverage on the soil temperature before the complete closure of the rice canopy (Figures S1 and S2) may decrease the nutrient uptake rate by the roots through a variety of chemical, physical, and biological responses to temperature [35]. The concentrations of essential nutrients, except for P and Fe, were significantly decreased in the rice seedlings (Table S2), but the magnitudes of the reductions varied for different elements. It is likely that the observed responses for each element are driven by a complex interaction involving rice-plant and soil responses to the duckweed treatments, necessitating more research to fully understand the underlying mechanisms.

During the period of the rice canopy closure (from 27 July to 11 August), rapid duckweed senescence and decomposition caused great changes in the soil’s biochemical properties (Figures S1–S3). The cooling effect of the duckweed coverage on the soil temperature disappeared at the complete closure of the rice canopy (Figures S1 and S2). However, a substantial further decrease in Eh was observed at the complete closure of the rice canopy in the duckweed-covered plots (Figure S3), indicating that a great amount of duckweed decomposition further decreased the soil’s redox potential. A low Eh value may promote the formation of insoluble ZnS and CuS [36,37], thereby decreasing the concentrations of the free ions of Zn^2+^ and Cu^2+^ in the soil solution; this may result in low uptakes of these ions by the rice.

## 4. Materials and Methods

### 4.1. Experimental Site

This study was conducted at Yangzhou University in Yangzhou (119°42′0″ E, 32°35′5″ N), Jiangsu Province, China. Yangzhou belongs to the subtropical monsoon climate, with an average annual temperature of 14.8 °C, an average annual precipitation of 1020 mm, and an annual sunshine time of 2000 h. Concrete tanks (8.4 m × 1.4 m × 0.4 m) were filled with lotus-pond bottom soil, which had the following properties: pH 7.3; available nitrogen (N), 160.9 mg kg^−1^; available phosphorus (P), 47.3 mg kg^−1^; available potassium (K), 209.5 mg kg^−1^; available copper (Cu), 26.0 mg kg^−1^; available iron (Fe), 135.2 mg kg^−1^; available manganese (Mn), 18.8 mg kg^−1^; and available Zinc (Zn), 26.3 mg kg^−1^. This growth medium contained high levels of mineral nutrients and was used to simulate the situation of an organic fertilizer application that caused a duckweed outbreak in paddy fields.

### 4.2. Treatments and Crop Cultivation

Each concrete tank was divided into two parts from the middle: half of the tank was covered by duckweed and the other half was duckweed free as a control. The duckweed treatment started at the time of the rice transplantation. In the current investigation, three conventional *Japonica* cultivars, Yangnongxiang 28 (YNX28, released in 2020); Nanjing 9108 (NJ9108, released in 2013); and Jinxiangyu 1 (JXY1, released in 2020), were used. These three rice cultivars are widely grown in the Yangtze River region and favored by local farmers because of their high quality and yield stability. Rice seeds were sown in a nursing paddy on 15 May 2022. All the rice seedlings were manually transplanted at a spacing of 19.5 × 17.5 cm into concrete tanks filled with lotus-pond bottom soil after 36 days of germination at a density of 2 seedlings per hill. The soil was submerged with water at a level of about 5 cm in depth from transplantation to harvest. To maintain normal plant growth and development, pests, diseases, and weeds were controlled. All the cultivars reached the 50% heading stage from mid to late August, and the maturity date was from mid to late October.

### 4.3. Parameter Measurements

The soil temperatures and Eh values were measured at 16:30–17:30 at each measurement date. The soil temperatures at depths of 5 cm and 10 cm were measured with a thermometer every two weeks from 1 July to 25 August. The soil Eh value at a depth of 5 cm was measured with an Eh meter every two weeks from 12 July to 13 October.

At the tillering stage (5 July), 3 hills of the plants were selected from each treatment for each cultivar to determine the element concentrations in the shoots. For the determination of the N concentration, a dry-pulverized sample of the shoot was digested with concentrated sulfuric acid at 370 °C, and the N concentration of the filtrate was determined using a discrete auto analyzer (Smartchem200, AMS-Westco, Rome, Italy), the colorimetric method, and salicylate. The mineral concentrations in the shoots were determined as follows: First, shoot samples were dried to a constant mass and ground to a fine powder. Then, 0.2 g of each sample was ashed in a muffle furnace for 6 h at 500 °C. The samples were dissolved in a 6% HNO_3_ solution and filtered through quantitative filter papers. We used an inductively coupled plasma emission spectrometer (ICP-AES, iCAP6300, Thermo Fisher Scientific, Waltham, MA, USA) to determine the mineral element concentrations in the filtrate. We measured the contents of the macroelements P, K, Ca, and Mg along with those of the microelements Fe, Mn, Cu, and Zn. The green-tea standard reference material (GBW100721) was used to ensure the precision of the analytical procedures.

At maturity (from mid to late October), 3 hills of the plants were harvested from each plot for each cultivar to determine the rice yield and quality. The grain was threshed manually and divided into empty, fully filled, and incompletely filled grains. Subsequently, the grain weight and number were obtained. The yield and its traits were calculated as follows: filled-grain percentage (%) = number of filled grains/total number of spikelets × 100%; filled-grain weight (mg) = the total weight of filled-grains (mg)/number of filled grains; yield (g plant^−1^) = number of panicles × number of spikelets per panicle × filled-grain percentage × filled-grain weight (mg)/1000. Subsequently, the quality traits were determined according to the China National Standard GB/T 17891-2017 issued by the General Administration of Quality Supervision, Inspection, and Quarantine and the Standardization Administration of the People’s Republic of China. The evaluation of the rice-processing quality included the brown-rice percentage, milled-rice percentage, and head-rice percentage. The grains were dehulled to produce brown rice, using a chaff-remover machine, and the brown-rice percentage was determined. The brown rice was divided into two parts: one for the elemental composition determination and the other for the determination of the other qualities after further processing. Then, the brown rice was milled with a rice-polishing machine (Pearlest, Kett, Tokyo, Japan) until a standard degree of milling was reached for evaluating the milling properties (i.e., milled-rice percentage and head-rice percentage). The appearance quality characteristics (i.e., the chalky-grain percentage, chalkiness degree, grain length, grain width, and ratio of the grain length to the grain width) of head rice were measured using a chalkiness visualization scanner equipped with JMWT12 software (Dongfujiuheng, Beijing, China).

After the evaluation of the appearance, the milled rice samples were ground into a powder by a vibration disk mill (TS1000, Siebtechnik GmbH, Mülheim, Germany) to further prepare them for subsequent analyses. The starch viscosity of the milled rice and taste score of the cooked rice were determined according to the methods developed by Gao et al. [38].

The protein concentration in the brown rice was measured using an Infratec™ 1241 grain analyzer (FOSS Tecator, Hillerod, Denmark), which is a near-infrared transmittance (NIT) analyzer. The mineral concentrations in the brown rice were determined using ICP-AES (iCAP6300, Thermo Fisher Scientific, Waltham, MA, USA) after the brown-rice samples were digested using a microwave digestion system (MARS5, CEM Corporation, Matthews, NC, USA) [38]. The wheat-standard reference material (GBW080684) was used to ensure the precision of the analytical procedures.

### 4.4. Statistical Analyses

The experiment represented a completely randomized design with three replicates. Analysis of variance (ANOVA) for the treatments, cultivars, and their interactions were performed using SPSS statistical software (SPSS 26.0, IBM Company, Chicago, IL, USA). The data in the tables and figures are in the form of mean values ± standard errors. Statistically significant effects were indicated as follows: ** *p* < 0.01; * *p* < 0.05.

## 5. Conclusions

Duckweed coverage during rice growth increased the protein concentration but decreased the essential micronutrient concentrations in rice grains. This preliminary investigation highlights the need for further research into the responses of the rice quality to the duckweed coverage. First, it remains to be determined how broadly applicable our results are outside the pond-bottom soil and rice cultivars used in this experiment. The other rice cultivars grown in paddy fields with limited fertility may respond differently to duckweed, with unknown effects on the nutritional value of rice grains. Second, the potential for duckweed acting as a “nutrient buffer” to retain N in paddies, resulting in a continuous N supply to rice plants at the late rice-growth stage is an attractive hypothesis for further testing.

## Figures and Tables

**Table 1:** Effects of duckweed coverage on grain yield and yield components of different rice cultivars.

Cultivar	Treatment	Panicle Number per Plant	Spikelet Number per Panicle	Filled-Grain Percentage (%)	Grain Weight (mg)	Grain Yield per Plant (g)
JXY1	Control	13.3 ± 0.5	202.6 ± 4.1	56.9 ± 1.2	19.5 ± 0.1	29.8 ± 0.5
	Duckweed	11.7 ± 0.6	240.1 ± 10.5	55.7 ± 0.9	20.2 ± 0.1	31.6 ± 1.4
NJ9108	Control	10.3 ± 0.8	182.1 ± 10.9	69.8 ± 5.1	20.8 ± 0.2	27.2 ± 2.3
	Duckweed	9.8 ± 0.8	190.9 ± 4.8	58.5 ± 3.4	21.0 ± 0.2	22.9 ± 1.3
YNX28	Control	11.1 ± 0.5	181.1 ± 6.7	72.7 ± 2.1	20.0 ± 0.2	29.3 ± 2.0
	Duckweed	9.7 ± 0.3	181.4 ± 4.1	68.9 ± 4.2	20.4 ± 0.1	24.7 ± 1.7
All	Control	11.5 ± 0.5	188.6 ± 5.2	66.5 ± 2.9	20.1 ± 0.2	28.8 ± 1.0
	Duckweed	10.4 ± 0.4	204.1 ± 9.8	61.0 ± 2.6	20.5 ± 0.1	26.4 ± 1.5
ANOVA results						
Duckweed		0.033	0.025	0.061	0.007	0.103
Cultivar		0.002	<0.001	0.003	<0.001	0.015
Duckweed × Cultivar		0.645	0.065	0.294	0.210	0.130

Values are means ± standard errors.

**Table 2:** Effects of duckweed coverage on various traits of rice-grain processing and appearance qualities of different rice cultivars.

Cultivar	Treatment	Brown-Rice Percentage (%)	Milled-Rice Percentage (%)	Head-Rice Percentage (%)	Chalky-Grain Percentage (%)	Chalkiness Degree (%)	Green-Grain Percentage (%)	Head-Rice Length (mm)	Head-Rice Width (mm)	Length–Width Ratio (L/W)
JXY1	Control	84.4 ± 0.5	77.0 ± 0.6	69.6 ± 1.0	72.2 ± 0.3	39.0 ± 0.3	2.0 ± 1.2	4.07 ± 0.02	2.53 ± 0.01	1.61 ± 0.01
	Duckweed	85.1 ± 0.1	77.4 ± 0.0	62.3 ± 4.2	79.1 ± 1.3	45.2 ± 0.8	3.6 ± 0.6	4.13 ± 0.01	2.57 ± 0.02	1.61 ± 0.01
NJ9108	Control	83.5 ± 0.4	76.5 ± 0.2	70.6 ± 2.3	67.3 ± 3.7	43.6 ± 2.4	1.1 ± 1.0	4.13 ± 0.03	2.62 ± 0.02	1.57 ± 0.00
	Duckweed	83.7 ± 0.6	76.9 ± 0.4	61.2 ± 2.9	62.3 ± 1.6	38.3 ± 1.4	3.7 ± 1.6	4.12 ± 0.02	2.62 ± 0.01	1.57 ± 0.01
YNX28	Control	84.0 ± 0.1	76.9 ± 0.2	52.2 ± 4.0	71.7 ± 0.9	42.2 ± 0.8	5.3 ± 1.5	4.25 ± 0.03	2.48 ± 0.02	1.71 ± 0.01
	Duckweed	84.1 ± 0.1	76.9 ± 0.2	63.3 ± 3.9	78.4 ± 1.2	43.4 ± 0.8	6.5 ± 1.4	4.30 ± 0.01	2.50 ± 0.01	1.72 ± 0.01
ALL	Control	84.0 ± 0.2	76.8 ± 0.2	64.1 ± 3.3	70.4 ± 1.4	41.6 ± 1.0	2.8 ± 0.9	4.15 ± 0.03	2.54 ± 0.02	1.63 ± 0.02
	Duckweed	84.3 ± 0.3	77.1 ± 0.2	62.3 ± 1.9	73.3 ± 2.8	42.3 ± 1.2	4.6 ± 0.8	4.18 ± 0.03	2.56 ± 0.02	1.63 ± 0.02
ANOVA results										
Duckweed		0.276	0.394	0.498	0.079	0.514	0.101	0.056	0.145	0.867
Cultivar		0.022	0.351	0.040	<0.001	0.378	0.031	<0.001	<0.001	<0.001
Duckweed × Cultivar		0.746	0.811	0.015	0.010	0.003	0.839	0.194	0.440	0.636

Values are means ± standard errors.

**Table 3:** Effects of duckweed coverage on the RVA profiles of milled rice flours from different rice cultivars.

Cultivar	Treatment	Peak Viscosity (cP)	Hot Viscosity (cP)	Breakdown (cP)	Final Viscosity (cP)	Setback (cP)	Peak Time (min)	Pasting Temperature (°C)	Consistency (cP)
JXY1	Control	2467.0 ± 142.0	1149.7 ± 108.9	1317.3 ± 36.6	2034.8 ± 123.5	−432.2 ± 21.3	5.8 ± 0.0	73.5 ± 0.5	885.2 ± 15.6
	Duckweed	2472.7 ± 117.0	1262.3 ± 85.4	1210.3 ± 36.7	2089.0 ± 121.5	−383.7 ± 10.4	5.9 ± 0.0	74.0 ± 0.0	826.7 ± 37.8
NJ9108	Control	2291.8 ± 136.3	1060.5 ± 84.4	1231.3 ± 86.6	1892.0 ± 114.3	−399.8 ± 61.6	5.7 ± 0.1	73.1 ± 0.5	831.5 ± 35.4
	Duckweed	2285.3 ± 41.1	1105.3 ± 18.2	1180.0 ± 40.5	1917.0 ± 21.2	−368.3 ± 34.8	5.8 ± 0.0	73.7 ± 0.3	811.7 ± 6.6
YNX28	Control	2921.0 ± 35.5	1233.0 ± 33.9	1688.0 ± 24.2	2016.3 ± 33.2	−904.7 ± 19.0	5.6 ± 0.0	73.1 ± 0.0	783.3 ± 5.2
	Duckweed	2972.7 ± 13.7	1375.0 ± 31.8	1597.7 ± 18.1	2171.3 ± 38.0	−801.3 ± 24.5	5.8 ± 0.1	73.1 ± 0.1	796.3 ± 8.2
ALL	Control	2559.9 ± 110.1	1147.7 ± 47.9	1412.2 ± 75.5	1981.1 ± 54.4	−578.9 ± 83.9	5.7 ± 0.0	73.2 ± 0.2	833.3 ± 18.5
	Duckweed	2576.9 ± 108.7	1247.6 ± 47.4	1329.3 ± 69.3	2059.1 ± 52.8	−517.8 ± 72.0	5.8 ± 0.0	73.6 ± 0.2	811.6 ± 12.1
ANOVA results									
Duckweed		0.833	0.103	0.048	0.297	0.043	0.004	0.195	0.261
Cultivar		<0.001	0.025	<0.001	0.109	<0.001	0.012	0.116	0.040
Duckweed × Cultivar		0.951	0.776	0.828	0.744	0.541	0.770	0.549	0.320

Values are means ± standard errors.

**Table 4:** Effects of duckweed coverage on the palatability of cooked rice from different rice cultivars.

Cultivar	Treatment	Overall Palatability Index	Luster	Hardness	Stickiness	Balance Degree
JXY1	Control	47.0 ± 0.6	3.6 ± 0.1	7.9 ± 0.1	2.6 ± 0.1	2.9 ± 0.1
	Duckweed	44.0 ± 2.0	3.1 ± 0.3	8.1 ± 0.2	2.2 ± 0.3	2.5 ± 0.3
NJ9108	Control	43.0 ± 0.6	3.0 ± 0.1	8.3 ± 0.1	2.0 ± 0.1	2.2 ± 0.1
	Duckweed	45.0 ± 1.0	3.3 ± 0.2	8.2 ± 0.2	2.4 ± 0.2	2.5 ± 0.2
YNX28	Control	45.7 ± 1.8	3.3 ± 0.3	8.0 ± 0.2	2.3 ± 0.2	2.7 ± 0.3
	Duckweed	46.3 ± 2.6	3.3 ± 0.4	8.1 ± 0.3	2.6 ± 0.3	2.7 ± 0.4
ALL	Control	45.2 ± 0.8	3.3 ± 0.1	8.1 ± 0.1	2.3 ± 0.1	2.6 ± 0.1
	Duckweed	45.1 ± 1.1	3.2 ± 0.2	8.1 ± 0.1	2.4 ± 0.1	2.6 ± 0.2
ANOVA results						
Duckweed		0.934	0.730	0.617	0.484	0.960
Cultivar		0.457	0.719	0.531	0.445	0.403
Duckweed × Cultivar		0.310	0.382	0.673	0.153	0.379

Values are means ± standard errors.

**Table 5:** Effects of duckweed coverage on element concentrations in brown rice for different rice cultivars.

Cultivar	Treatment	Protein (mg g^−1^)	P (mg g^−1^)	K (mg g^−1^)	Ca (mg g^−1^)	Mg (mg g^−1^)	Fe (mg kg^−1^)	Mn (mg kg^−1^)	Cu (mg kg^−1^)	Zn (mg kg^−1^)
JXY1	Control	115.33 ± 0.67	3.64 ± 0.05	2.43 ± 0.05	0.16 ± 0.01	1.47 ± 0.02	10.87 ± 0.83	22.50 ± 0.26	1.53 ± 0.07	19.42 ± 1.99
	Duckweed	121.67 ± 2.19	3.74 ± 0.15	2.66 ± 0.03	0.16 ± 0.01	1.44 ± 0.05	10.89 ± 0.12	17.04 ± 0.21	1.04 ± 0.15	15.96 ± 1.60
NJ9108	Control	121.33 ± 0.67	3.89 ± 0.17	2.49 ± 0.14	0.19 ± 0.02	1.61 ± 0.06	13.13 ± 0.40	17.72 ± 0.56	1.62 ± 0.25	25.33 ± 1.70
	Duckweed	126.67 ± 0.88	3.69 ± 0.09	2.47 ± 0.07	0.19 ± 0.04	1.46 ± 0.03	11.97 ± 0.83	17.82 ± 1.08	0.83 ± 0.04	17.08 ± 0.52
YNX28	Control	111.67 ± 1.86	3.51 ± 0.16	2.55 ± 0.14	0.21 ± 0.02	1.46 ± 0.09	11.71 ± 1.11	17.64 ± 0.13	1.45 ± 0.14	20.74 ± 2.78
	Duckweed	114.67 ± 2.33	3.24 ± 0.05	2.37 ± 0.03	0.15 ± 0.01	1.34 ± 0.01	10.06 ± 0.44	18.01 ± 1.27	1.20 ± 0.20	19.68 ± 1.91
ALL	Control	116.11 ± 1.53	3.68 ± 0.09	2.49 ± 0.06	0.19 ± 0.01	1.51 ± 0.04	11.91 ± 0.53	19.29 ± 0.82	1.53 ± 0.09	21.83 ± 1.42
	Duckweed	121.00 ± 1.99	3.56 ± 0.10	2.50 ± 0.05	0.17 ± 0.01	1.41 ± 0.03	10.97 ± 0.39	17.62 ± 0.51	1.02 ± 0.09	17.57 ± 0.92
ANOVA results										
Duckweed		0.003	0.247	0.918	0.321	0.033	0.130	0.017	0.002	0.017
Cultivar		<0.001	0.012	0.639	0.304	0.044	0.055	0.029	0.824	0.196
Duckweed × Cultivar		0.579	0.296	0.119	0.308	0.434	0.496	0.003	0.280	0.191

Values are means ± standard errors.

**Table 6:** Effects of duckweed coverage on element contents in brown rice for different rice cultivars.

Cultivar	Treatment	Protein (g m^−2^)	P (g m^−2^)	K (g m^−2^)	Ca (g m^−2^)	Mg (g m^−2^)	Fe (mg m^−2^)	Mn (mg m^−2^)	Cu (mg m^−2^)	Zn (mg m^−2^)
JXY1	Control	72.55 ± 1.23	2.29 ± 0.04	1.53 ± 0.05	0.10 ± 0.01	0.92 ± 0.01	6.85 ± 0.62	14.15 ± 0.18	0.96 ± 0.03	12.19 ± 1.14
	Duckweed	81.76 ± 3.27	2.53 ± 0.21	1.79 ± 0.11	0.11 ± 0.01	0.97 ± 0.07	7.32 ± 0.26	11.47 ± 0.60	0.70 ± 0.11	10.72 ± 1.09
NJ9108	Control	68.91 ± 5.82	2.22 ± 0.26	1.42 ± 0.19	0.11 ± 0.01	0.92 ± 0.11	7.46 ± 0.68	10.01 ± 0.60	0.90 ± 0.08	14.23 ± 0.48
	Duckweed	60.67 ± 3.03	1.77 ± 0.11	1.18 ± 0.06	0.09 ± 0.02	0.70 ± 0.04	5.78 ± 0.68	8.56 ± 0.84	0.40 ± 0.04	8.21 ± 0.64
YNX28	Control	68.58 ± 3.77	2.15 ± 0.08	1.56 ± 0.03	0.13 ± 0.02	0.89 ± 0.05	7.30 ± 1.22	10.86 ± 0.80	0.90 ± 0.14	12.66 ± 1.50
	Duckweed	59.41 ± 2.93	1.68 ± 0.12	1.23 ± 0.07	0.08 ± 0.01	0.69 ± 0.05	5.21 ± 0.33	9.41 ± 1.14	0.63 ± 0.13	10.34 ± 1.60
ALL	Control	70.01 ± 2.13	2.22 ± 0.08	1.50 ± 0.06	0.11 ± 0.01	0.91 ± 0.03	7.20 ± 0.45	11.68 ± 0.70	0.92 ± 0.05	13.03 ± 0.64
	Duckweed	67.28 ± 3.94	1.99 ± 0.15	1.40 ± 0.11	0.09 ± 0.01	0.79 ± 0.05	6.10 ± 0.39	9.81 ± 0.62	0.58 ± 0.07	9.76 ± 0.71
ANOVA results										
Duckweed		0.372	0.108	0.231	0.117	0.029	0.079	0.010	0.001	0.004
Cultivar		0.005	0.019	0.010	0.933	0.057	0.516	0.001	0.198	0.966
Duckweed × Cultivar		0.043	0.074	0.025	0.218	0.092	0.191	0.648	0.397	0.151

Values are means ± standard errors.

## Data Availability

The original data contributions presented in the study are included in the article/Supplementary Materials. Further inquiries can be directed to the Corresponding Authors.

## Supplementary Materials

The following supporting information can be downloaded at https://www.mdpi.com/article/10.3390/plants13010057/s1, Figure S1: Maximum air temperature and soil temperature at a depth of 5 cm; Figure S2: Maximum air temperature and soil temperature at a depth of 10 cm; Figure S3: Variation in Eh in soil at a depth of 5 cm; Table S1: Effects of duckweed coverage on grain yield and yield components in different experiments; Table S2: Effects of duckweed coverage on element contents of shoots in rice seedlings of different rice cultivars.
