# Supplementary material for: A Preliminary Study of the Impacts of Duckweed Coverage during Rice Growth on Grain Yield and Quality
Source: Plants (Basel). 2023 Dec 23;13(1):57. doi: 10.3390/plants13010057 (PMC10781075; doi:10.3390/plants13010057)
Supplement: Supplementary file 1 [file plants-13-00057-s001.zip › plants-2753214-supplementary.pdf]

A Preliminary Study of the Impacts of Duckweed Coverage during Rice Growth on Grain Yield and Quality

Jingsheng Luo [1,2], Shaowu Hu [3,4], Tong Li [1,2], Fuhao He [3,4], Chao Tian [3,4], Yu Han [3,4], Yulin Mao [3,4], Liquan Jing [3,4], Lianxin Yang [3,4,*] and Yunxia Wang [1,2,*]

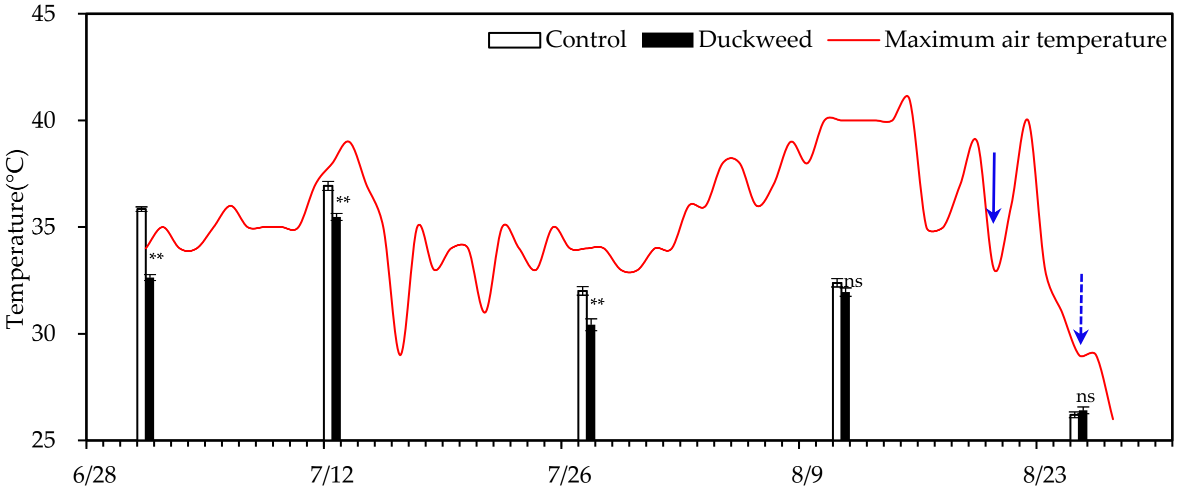

Fig. S1. Maximum air temperature and soil temperature at a depth of **5 cm**. Data are average values with ± standard error (vertical bars). *P<0.05, **P<0.01. Blue solid line with arrow: the heading date of rice under duckweed coverage; Blue broken line with arrow: the heading date of control rice.

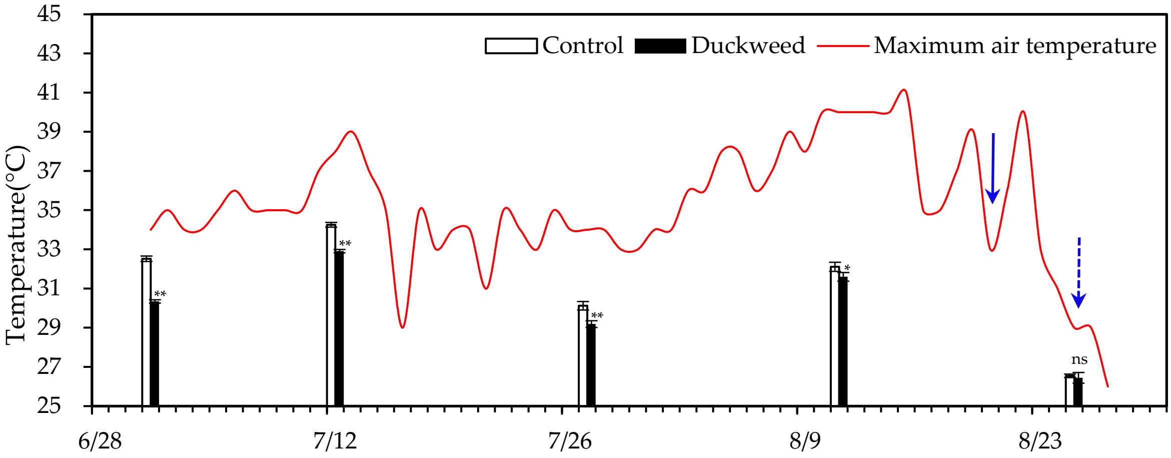

Fig. S2. Maximum air temperature and soil temperature at a depth of **10 cm**. Data are average values with ± standard error (vertical bars). **P*<0.05, ***P*<0.01. Blue solid line with arrow: the heading date of rice under duckweed coverage; Blue broken line with arrow: the heading date of control rice

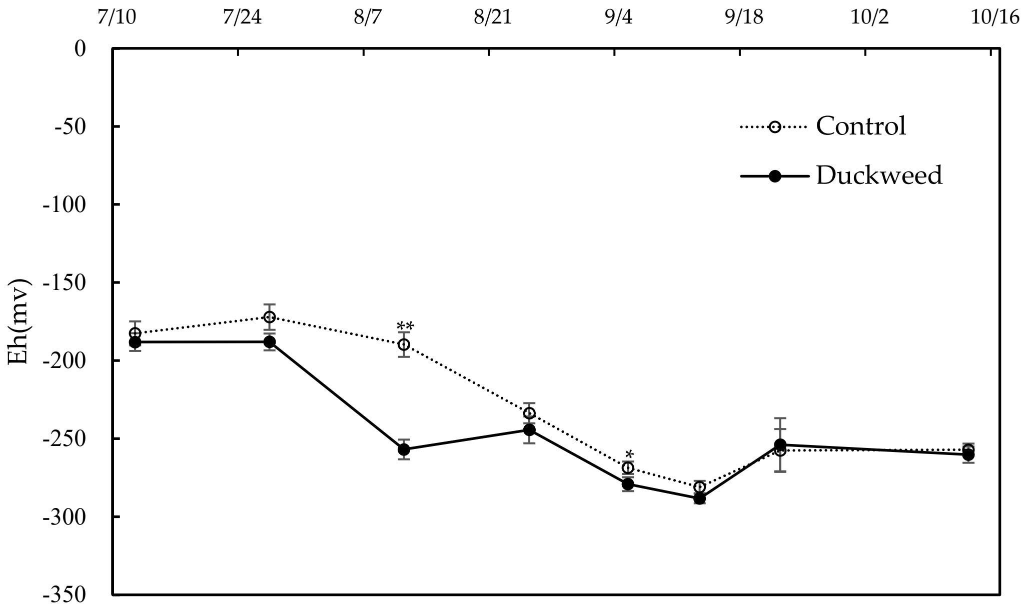

Fig. S3. Variation in Eh in soil at a depth of 5 cm. Data are average values with ± standard error (vertical bars). *P<0.05, **P<0.01.

Table S1. Effects of duckweed coverage on grain yield and yield components of different experiments.

Reference number	Treatments	Yield	Panicle density	Spikelet density	Filled grain percentage	Filled grain weight	Duckweed species	Rice species	Test years
[22]	1. Control<br>2. Urea<br>3. ENTEC (slow release fertilizer)<br>4. Biogas slurry<br>5. Control +duckweed<br>6. Urea+duckweed<br>7. ENTEC+duckweed<br>8. Biogas slurry+duckweed	↑ 8.6% ns (Control)<br>↑ 6.3% ns (ENTEC)<br>↑ 13.2% * (Urea)<br>↑ 16.9% * (Biogas slurry)					*Lemna minor L.*		2020
[26]	1. CK<br>2. *Landoltia punctata* coverage (LP)<br>3. *Spirodela polyrhiza* coverage (SP)	↑ 10.8% ns (LP)<br>↑ 23.7% * (SP)	↓ 1.0% ns (LP)<br>↓ 5.7% ns (SP)	↑ 4.2% ns (LP)<br>↑ 12.3% ns (SP)	↑ 12.0% ns (LP)<br>↑ 11.4% ns (SP)	↓ 1.1% ns (LP)<br>↑ 0.4% ns (SP)	1. *Landoltia punctata*<br>2. *Spirodela polyrhiza*		2018, 2019
[29]	Soils:<br>1. Hydragric Anthrosol<br>2. Haplic Acrisol<br>Treatments:<br>1. Urea+biochar<br>2. Urea+biochar+duckweed	Hydragric Anthrosol:<br>↑ 15.5% * (duckweed)<br>Haplic Acrisol:<br>↑ 6.7% ns (duckweed)							2017
[20]	1. 300 kg N/ha<br>2. 225 kg N/ha<br>3. 300 kg N/ha+duckweed<br>4. 225 kg N/ha+duckweed	2014:<br>↑ 11.0% * (225 kg N/ha)<br>↑ 14.6% * (300 kg N/ha)<br>2015:<br>↑ 15.1% * (225 kg N/ha)<br>↑ 14.0% * (300 kg N/ha)<br>2016:<br>↑ 14.3% * (225 kg N/ha)<br>↑ 7.9% * (300 kg N/ha)					*Spirodela polyrhiza*	Nanjing 46	2014, 2015, 2016
[19]	1. 0 kg N/ha<br>2. 90 kg N/ha<br>3. 180 kg N/ha<br>4. 0 kg N/ha+duckweed<br>5. 90 kg N/ha+duckweed<br>6. 180 kg N/ha+duckweed	↑ 4.3% * (0 kg N/ha)<br>↑ 10.8% * (90 kg N/ha)<br>↑ 8.7% * (180 kg N/ha)							2005
[14]A	1. 0% duckweed coverage<br>2. 25% duckweed coverage<br>3. 75% duckweed coverage<br>4. 100% duckweed coverage	↓ 23.5% (25% duckweed coverage)<br>↓ 25.9% (75% duckweed coverage)<br>↓ 34.6% (100% duckweed coverage)						Chengyou 489	2016
[27]	1. No fertilizer<br>2. Fertilizer<br>3. No fertilizer+Azolla pinnata<br>4. Fertilizer+Azolla pinnata	↓ 8.1% ns (No fertilizer)<br>↑ 18.2% ns (Fertilizer)					*Azolla pinnata*		2014, 2015
[28]	1. 0 kg N/ha<br>2. 0 kg N/ha+Azolla<br>3. 330 kg N/ha<br>4. 330 kg N/ha+Azolla<br>5. 280.5 kg N/ha<br>6. 280.5 kg N/ha+Azolla<br>7. 231 kg N/ha<br>8. 231 kg N/ha+Azolla	↑ 10.3% ns (0 kg N/ha)<br>↑ 2.9% ns (330 kg N/ha)<br>↑ 6.3% ns (280.5 kg N/ha)<br>↑ 7.7% ns (231 kg N/ha)<br>↑ ns (On average)					*Azolla filiculoides Lamarck*	Nanjing 9108	2019

Duckweed coverage increased(↑) , decreased(↓) , or had no significant effect(ns) on yield and yield components. *P<0.05. CK: Control. A, no statistic analysis was provided in this paper.

Table S2. Effects of duckweed coverage on element contents of shoots in rice seedlings of different rice cultivars.

Cultivars	Treatments	N (mg g^{-1})	P (mg g^{-1})	K (mg g^{-1})	Ca (mg g^{-1})	Mg (mg g^{-1})	Fe (mg kg^{-1})	Mn (mg kg^{-1})	Cu (mg kg^{-1})	Zn (mg kg^{-1})
JXY1	Control	33.72±0.72	4.95±0.54	23.63±0.20	5.84±1.79	2.84±0.29	315.95±103.41	444.81±13.24	2.14±0.26	11.59±0.52
	Duckweed	23.88±1.42	4.90±0.34	20.31±2.32	2.64±0.12	1.99±0.16	293.49±84.84	200.82±34.50	1.59±0.23	8.13±0.41
NJ9108	Control	32.64±1.09	4.72±0.21	25.11±1.92	3.59±0.43	2.18±0.05	390.00±41.02	435.83±75.01	1.70±0.05	14.14±0.46
	Duckweed	29.77±1.15	4.64±0.09	22.19±0.25	2.48±0.16	1.79±0.10	303.35±26.88	262.74±50.50	1.46±0.14	10.52±0.88
YNX28	Control	36.54±2.83	3.66±0.19	21.86±0.37	4.11±0.20	2.72±0.16	209.91±36.35	347.22±19.04	2.18±0.10	12.61±0.95
	Duckweed	30.77±1.42	4.61±0.18	20.22±0.95	3.90±0.43	2.09±0.15	278.46±102.37	246.02±22.98	1.65±0.06	10.08±0.55
All	Control	34.30±1.07	4.44±0.27	23.53±0.74	4.51±0.63	2.58±0.14	305.28±42.71	409.29±27.50	2.01±0.11	12.78±0.50
	Duckweed	28.14±1.26	4.72±0.12	20.91±0.79	3.01±0.26	1.96±0.08	291.77±39.32	236.53±21.01	1.57±0.08	9.58±0.49
ANOVA result										
Duckweed		<0.001	0.273	0.030	0.036	0.001	0.825	<0.001	0.006	<0.001
Cultivar		<0.001	0.055	0.170	0.301	0.041	0.399	0.473	0.119	0.010
Duckweed×Cultivar		0.127	0.183	0.801	0.188	0.413	0.581	0.270	0.564	0.680

Values are means ± standard errors.
